# Synthesis of superparamagnetic Fe_3_O_4_–graphene oxide-based material for the photodegradation of clonazepam

**DOI:** 10.1038/s41598-024-67352-8

**Published:** 2024-08-14

**Authors:** Maryne Patrícia da Silva, Ana Caroline Alves de Souza, Ágata Rodrigues Deodato Ferreira, Pedro Lucas Araújo do Nascimento, Tiago José Marques Fraga, Jorge Vinícius Fernandes Lima Cavalcanti, Marcos Gomes Ghislandi, Maurício Alves da Motta Sobrinho

**Affiliations:** 1 Department of Chemical Engineering, Federal University of Pernambuco (UFPE), 1235 Prof. Moraes Rego Av, Cidade Universitária, Recife, PE 50670-901 Brazil; 2 Department of Food Science, Federal University of Pernambuco Agreste (UFAPE), Bom Pastor Avenue, W/N, Boa Vista, Garanhuns, PE 55292-270 Brazil; 3 Federal Rural University of Pernambuco (UFRPE), 300 Cento e Sessenta e Três Av., Cabo de Santo Agostinho, PE Brazil

**Keywords:** Environmental sciences, Materials science, Nanoscience and technology

## Abstract

The global concern over water pollution caused by contaminants of emerging concern has been the subject of several studies due to the complexity of treatment. Here, the synthesis of a graphene oxide-based magnetic material (GO@Fe_3_O_4_) produced according to a modified Hummers’ method followed by a hydrothermal reaction was proposed; then, its application as a photocatalyst in clonazepam photo-Fenton degradation was investigated. Several characterization analyses were performed to analyze the structure, functionalization and magnetic properties of the composite. A 2^3^ factorial design was used for the optimization procedure to investigate the effect of [H_2_O_2_], GO@Fe_3_O_4_ dose and pH on clonazepam degradation. Adsorption experiments demonstrated that GO@Fe_3_O_4_ could not adsorb clonazepam. Photo-Fenton kinetics showed that total degradation of clonazepam was achieved within 5 min, and the experimental data were better fitted to the PFO model. A comparative study of clonazepam degradation by different processes highlighted that the heterogeneous photo-Fenton process was more efficient than homogeneous processes. The radical scavenging test showed that ${O}_{2}^{\cdot -}$ was the main active free radical in the degradation reaction, followed by hydroxyl radicals (^•^OH) and holes (h^+^) in the valence layer; accordingly, a mechanism of degradation was proposed to describe the process.

## Introduction

In a global context, the pharmaceutical industry has emerged as an agent that facilitates human life, representing today one of the largest industrial branches on the planet. However, its growth in recent decades has not kept pace with the evolution of effluent treatment techniques^1^. Contaminants of emerging concern from the pharmaceutical industry, the so-called Pharmaceutical Active Components (PhACs), have drawn the attention of world organizations for representing a risk to human health and the environment, affecting the quality of water consumed by the population^2^. This is because PhACs are synthesized to produce a certain biological response in a target organism; however, they can also produce the same response when in contact with another organism. Additionally, several studies have reported the presence of PhACs in water bodies around the world, even in restricted regions, such as the Antarctic, where there is no permanent human population. PhACs have very stable and complex structures, low volatility and different hydrophobicities. In this manner, there is an urgent need for more specific and effective techniques for the removal of PhACs from these effluents^3–7^.

Conventional treatment methods are not effective at efficiently removing this class of contaminants. As a result, various tertiary treatment techniques have been proposed for the remediation of these contaminants^5^. Adsorption has been expensively studied as an alternative treatment for the removal of this class of compounds in aquatic environments. In this regard, a wide variety of materials have been employed as adsorbents. Many are classified by the authors as low-cost; others are classified as materials with high adsorptive capacity and selectivity. However, the need to determine a treatment method for the contaminant adsorbed on the material surface, or for a disposal procedure for the adsorbent at the end of its useful life, has raised the question of whether adsorption would be an effective treatment method or merely a phase change of contaminants^8^. Advanced oxidative processes (AOPs) have been proven to be an alternative with great potential for PhAC removal. These processes mainly involve the generation of radicals such as hydroxyl radicals (^•^OH) and sulfate radicals (${SO}_{4}^{-\cdot}$) via the use of sound, light, electricity, high temperature and pressure^9,10^. These radicals act as “weak points” in pollutant molecules (conjugated acids, double bonds, heteroatoms, etc.), effectively breaking the molecular structure of the contaminant and causing its subsequent degradation^11^.

Among the types of AOPs, the Fenton process is based on the Fe-catalyzed decomposition of H_2_O_2_ into ^•^OH and stands out for its efficiency and low cost^2,12^, despite the generation of large amounts of sludge being considered a drawback. In heterogeneous Fenton reactions (*Fenton-like* reactions), a heterogeneous Fe catalyst is used to catalyze ^•^OH production on the surface. In addition, when irradiated, the catalyst will generate electrons and holes (e^−^ and h^+^) that can promote reduction and oxidation, respectively, and form new radicals. The heterogeneous Fenton process can be considered an alternative to the disadvantages of homogeneous processes because it is impossible to recover the catalyst and mitigate sludge production^13^. On the other hand, some of the e^−^/h^+^ pairs can recombine and reduce the process efficiency; additionally, the agglomeration of iron-based materials limits their efficiency^14–16^.

Graphene oxide (GO) has been applied as a carrier of metal oxides in AOP processes because of its good stability and high specific surface area. The oxygen-containing functional groups on the GO surface destroys the π bonds, reducing the ability of conducting electrons. In this sense, the reduction of GO to produces reduced graphene oxide (rGO) is a solution to repairs the electronic structure of the surface of GO. In this sense, GO and rGO can work as an electron acceptor, decreasing e^−^/h^+^ recombination and preventing the agglomeration of Fe nanoparticles, improving process performance^16–19^.

In the present work, the development of a graphene-based magnetic photocatalyst for the photo-Fenton degradation of clonazepam was proposed. GO was synthesized via the modified Hummers method. Subsequently, the material was functionalized to obtain GO@Fe_3_O_4_. A 2^3^ factorial design was used to determine the influence of pH, [H_2_O_2_] and photocatalyst dosage on the process. Adsorption and photodegradation kinetics were performed, and based on these results, a mechanism was proposed. A comparative study was carried out to relate the efficiency of the process proposed in this work to the efficiency of homogeneous processes, adsorptive processes and other types of radiation sources for the degradation of the same contaminant under the same conditions.

## Methodology

### Basic contaminant information and bench UV-C reactor model

**Figure 1:**
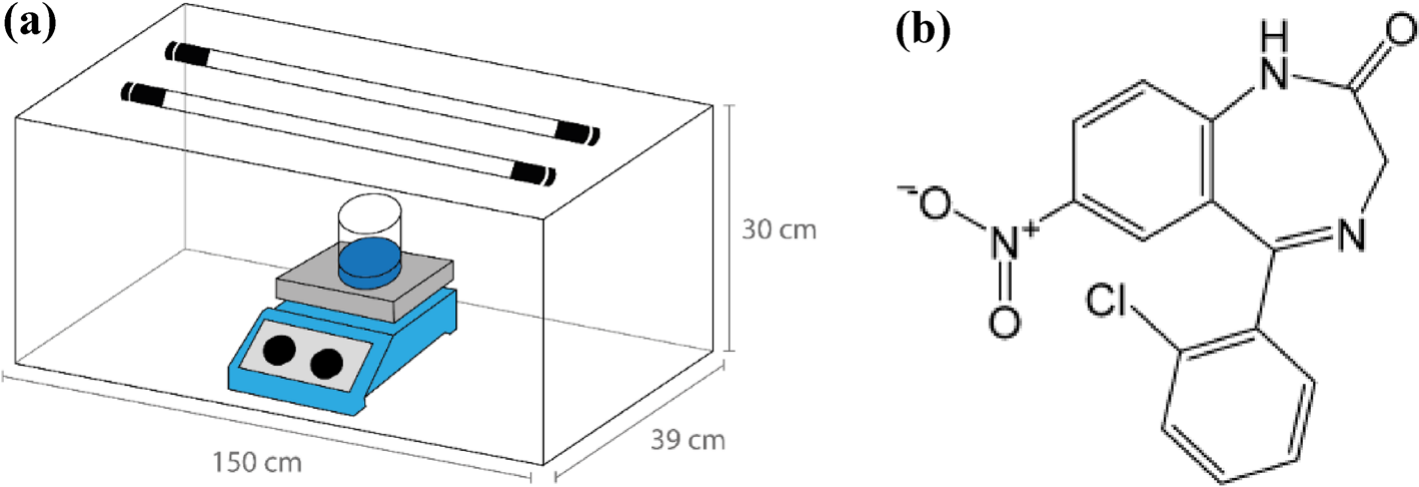
(**a**) Representative scheme of the photoreactor used in the tests; (**b**) Molecular formula of clonazepam.

The experiment was performed using a bench reactor equipped with two UV‒C lamps (Philips, TUV model, 30 W 1SL/25) coupled to a magnetic stirrer where the Petri dishes were placed (Fig. 1a). Clonazepam (CZP) (Fig. 1b) [5-(2-chlorophenyl)-1, 3dihydro-7-nitro-2H-1,4-benzodiazepin-2-one] is a pharmaceutical from a benzodiazepine series with a molecular weight equal to 315.715 g mol^−1^, a pKa_1_ value of 1.50 and a pKa_2_ value of 10.51^20,21^.

### Analytical method

The CZP concentration was measured using a high-performance liquid chromatography (HPLC) HP 1050 series Hewlett-Packard model equipped with a UV‒visible detector. Separation was performed on a Phenomenex Luna C18 (5 µm, 250 mm × 4.6 mm) column using methanol and distilled water (v/v = 70/30) as the mobile phase. The HPLC flow rate was 1 mL min^−1^, and the wavelength used was 254 nm.

### Graphene oxide synthesis and functionalization

GO was synthesized following a top-down method according to modified Hummers’ method employed in previous works^6,22^. For this purpose, 1.0 g of graphite powder was mixed with 25.0 mL of H_2_SO_4_ in a flask in an ice bath at a temperature less than 15.0 °C for 10 min. Then, 3.0 g of KMnO_4_ was slowly added to the system. The ice bath was removed, and the mixture was agitated at 200 rpm for 6 h. After 6 h, the oxidation was stopped using 35.0 mL of H_2_O_2_ and 200.0 mL of distilled water. The graphite oxide was washed with 5% HCl and distilled water. Then, graphite oxide was sonicated in an ultrasonicator (Elma, 40 kHz, model EM30H) for 4 h to obtain graphene oxide^23,24^.

The graphene oxide was functionalized by co-precipitation of Fe_3_O_4_ nanoparticles in the presence of GO. For this purpose, a previous aqueous solution of 100.0 mL of FeCl_3_·6H_2_O and FeSO_4_.7H_2_O was prepared at a 3:1 molar ratio. The iron solution and the GO were mixed at a 3:1 weight ratio under constant stirring (250 rpm) at room temperature (25.0 ± 5.0 °C). Then, the temperature was increased to 60.0–70.0 °C, and the pH was adjusted to 11.0 using ammonium hydroxide. The system was then isolated from the outside environment, and the stirring was continued for 6 h. Diverse washes were carried out with ethanol and distilled water until the pH stabilized^23,25^.

### Characterization of GO and GO@Fe_3_O_4_

Scanning electron microscopy (SEM) was used to analyze the surfaces of Fe_3_O_4_, GO and GO@Fe_3_O_4_ using a Tescan model Vega 3. Energy dispersive spectroscopy (EDS) was carried out with an Aztec Live spectrometer (Oxford) attached to the SEM equipment. All the samples were covered with gold under vacuum prior to SEM/EDS analysis.

Optical properties were measured by UV‒Vis diffuse reflectance spectroscopy (DRS) in the range of 200–800 nm. The analysis was performed with a Shimadzu 2700 model equipped with an ISR-2600 integrating sphere unit. The optical band-gap energy was measured following the Kubelka–Munk function, according to Eq. (1), and plotting [F(R) × hν]^2^ versus hν.

1 $$F\left(R\right)=\frac{{(1-R)}^{2}}{2R}$$

where R is the reflectance.

X-ray photoelectron spectroscopy (XPS) was performed on a ThermoScientific spectrometer (model Alpha, hυ = 1486.6 eV) to investigate the groups attached to the surface of the GO@Fe_3_O_4_. The XPS deconvolutions in the high-resolution were analyzed using the CasaXPS software with Gaussian (Y%)-Lorentzian (X%) profiles. The background from each spectrum was subtracted using Shirley-type background. The deconvolutions in the high-resolution spectrum were fitted with least chi-square value and an R^2^ value of 0.99.

From the results obtained by XPS analysis, a quantitative analysis of the surface elemental composition was performed using an empirical approach of the measured areas according to Eq. 2^26,27^.

2 $${x}_{i}=\frac{{A}_{i}/{RSF}_{i}}{{\sum }_{j=1}^{n}\left({A}_{j}/{RSF}_{j}\right)}$$

where A_i_ is the area under the corresponding core-level peak, and RSF_i_ is the relative sensitivity factor.

Fourier-transformed infrared spectroscopy (FTIR) was used to determine the functional groups of Fe_3_O_4_ and GO@Fe_3_O_4_. For this purpose, an IR Prestige-21 spectrometer (Shimadzu) was used, and all the samples were subjected to infrared emission through attenuated total reflectance (ATR) crystal analysis.

The crystallographic structures of the nanocomposites were analyzed using X-ray diffraction (XRD). The analysis was performed with a D8 ADVANCE diffractometer (Bruker) equipped with a copper radiation source (λ = 1.5418 Å).

The pH of zero-point of charge (pH_ZPC_) was determined by the salt addition method. For this purpose, 0.1 g of GO@Fe_3_O_4_ was mixed with 40.0 mL of NaCl for 24 h. The pH of the suspension was previously adjusted to an initial value of 2, 3, 4, 5, 6, 7, 8, 9, 10, 11 and 12 using HCl and NaOH 1.0 mol L^−1^ solutions and a Quimis pHmeter model Q400 as^25^.

The magnetization property of GO@Fe_3_O_4_ was investigated using a vibrating sample magnetometer (VSM), model Av 7 (Microsense).

### 2^3^ Factorial design

To determine the ideal initial conditions, a 2^3^ factorial design with a central point in triplicate was conducted. For this purpose, the mass of GO@Fe_3_O_4,_ the initial pH of the solution and the concentration of hydrogen peroxide were investigated. The experiments were performed using 80.0 mL of a CZP solution with an initial concentration of 6.0 mg L^−1^. The values of the parameters were as follows: m[GO@Fe_3_O_4_]: 1 mg (−), 5.5 mg (0), and 10 mg (+); [H_2_O_2_]: 15.0 mg L^−1^ (−), 33.0 mg L^−1^ (0), 51.0 mg L^−1^ (+); pH: 3 (−), 6 (0), 9 (+). The choice of H_2_O_2_ concentration was based on a COD of 20.0 mg L^−1^ clonazepam solution. The combination of each parameter was realized following Table S1 (Supplementary Material), and the experiments were performed randomly to avoid unusual deviations associated with certain combinations of levels. The system was placed in a UV-C reactor for 15 min. The statistical analysis was performed using the software STATISTICA 12 and the “pure error” model.

### Adsorption and photodegradation kinetics

The adsorption capacity of GO@Fe_3_O_4_ was investigated to determine, at the end of the process, the percentage of removal due to adsorption and photodegradation. For this purpose, the kinetics of adsorption were determined using 80.0 mL of a solution of CZP 6.0 mg L^−1^ of concentration. The experiment was performed without any source of radiation or H_2_O_2_. Therefore, 1 mg of GO@Fe_3_O_4_ was put in the system with the drug solution, and the final concentration was measured at the end of the following steps: 1, 3, 5, 7, 10, 13, 15, 20 and 30 min. The final concentration was measured by an HPLC-chromatographer, and the adsorptive capacity was calculated using Eq. (3).

3 $${\text{q}}_{\text{t}} = \frac{\left({\text{C}}_{0}-{\text{C}}_{\text{f}}\right)\cdot {\text{V}}}{{\text{m}}}$$

where q_t_ (mg g^−1^) is the adsorptive capacity; C_0_ and C_f_ are the initial and final concentrations of clonazepam (mg L^−1^), respectively; V is the volume of contaminant solution (L); and m is the dosage of the material (g).

After the adsorption kinetic, the time of equilibrium was identified and before the photodegradation kinetic, the photocatalyst was mixed with 80.0 mL of a solution of clonazepam (6.0 mg L^−1^) without radiation for a time equal to the time of equilibrium. Then, the concentration was measured and considered as the initial concentration for photodegradation kinetic. Then, H_2_O_2_ was added to the system at a concentration of 15.0 mg L^−1^ and the UV-C reactor was turned on. The kinetics were determined according to the adsorption kinetic, and at the end, the final concentration was measured via an HPLC-chromatographer. The data were fitted for the PFO model proposed by^28^ (Eq. (4)), PSO (Eq. (5)) and Chan & Chu model (Eq. (6)). All experiments were conducted in duplicate.

4 $$\frac{{\text{C}}_{\text{t}}}{{\text{C}}_{0}} = {\text{e}}^{-{\text{k}}_{1}{\text{t}}}$$

5 $$\frac{{\text{C}}_{\text{t}}}{{\text{C}}_{0}} = \frac{1}{{\text{k}}_{2}{{\text{C}}}_{\text{o}}{\text{t}}+1}$$

6 $$\frac{{\text{C}}_{\text{t}}}{{\text{C}}_{0}} = {1}-\frac{\text{t}}{{(\uprho+\upsigma{\text{t}})}}$$

In which C_0_ (mg L^−1^) is the initial concentration of the clonazepam solution; C_t_ (mg L^−1^) is the concentration of clonazepam solution at time t; k_1_ (min^−1^) is the apparent first-order rate constant; k_2_ (L mg^−1^ min^−1^) is the apparent second-order rate constant; t (min) is the time; 1/ρ (min^−1^) is the initial removal rate of clonazepam; and 1/σ (dimensionless) is the maximum oxidative capacity in the process.

### Comparison of different processes in clonazepam degradation and radical scavenging tests

The efficiency of the photo-Fenton reaction with GO@Fe_3_O_4_ under UV-C radiation was compared with that of other processes, including homogeneous processes; for not necessarily, a photocatalyst is sometimes less expensive than a heterogeneous process. In addition, other radiation sources, such as UV-A (Taschibra TF40T-811y, 120.0 cm, 40 W, 1.72 W m^−2^) and LED (Black-Decker BDT8-0900, 87.0 cm, 10.0 W, 15.60 W m^−2^ at 550 nm), were tested. The experiment was carried out under identical experimental conditions (pH 3.0; T = 25.0 °C; GO and GO@Fe_3_O_4_ dosage = 1 mg; [H_2_O_2_] = 15.0 mg L^−1^) for 10 min.

The radical scavenging test was evaluated to obtain information about the degradation mechanism. For this purpose, benzoquinone (BZQ), formic acid (FA) and methanol were used to trap superoxide radical anions (${O}_{2}^{\cdot -}$), holes (h^+^) and hydroxyl radicals (^•^OH), respectively. The experimental conditions were the same as those used in the kinetic study, and the concentration of the scavenger was 0.1 M.

## Results and discussion

### Characterization of GO and GO@Fe_3_O_4_

**Figure 2:**
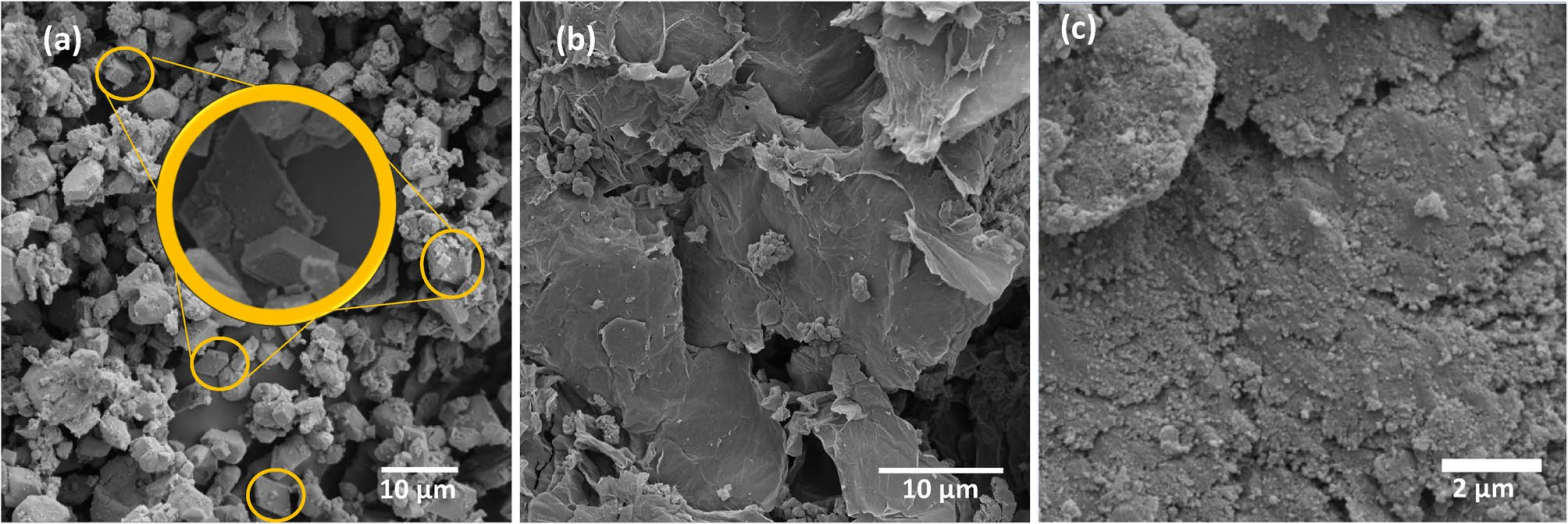
SEM images of Fe_3_O_4_ (**a**), GO (**b**) and GO@Fe_3_O_4_ (**c**).

Scanning electron microscopy (SEM) analysis was performed to assess the morphology of the materials. Figure 2a shows an SEM image of the Fe_3_O_4_ nanoparticles, where it is possible to observe small particles (< 5 µm) with a tendency toward octahedral morphology, which is attributed to the use of NH_4_OH. Previous works have shown the influence of the NH_4_OH concentration on the morphology and magnetic properties of magnetite^29^. However, their heterogeneity may be due to the use of concentrated NH_4_OH. A study conducted by Perez et al. (2020) showed that more heterogeneous conditions in terms of shape and size of Fe_3_O_4_ particles were obtained when concentrated NH_4_OH was used. On the other hand, more homogeneous shapes were obtained when NH_4_OH was used in conjunction with NaNO_3_^29^. Figure 2a also shows the agglomeration of the particles that occurs due to their strong anisotropic dipolar interactions^30^.

Figure 2c shows the surface morphology of GO@Fe_3_O_4,_ in which a rough and wrinkled agglomerations resembling a spongier structure could be observed; these agglomerations were not observed on the GO surface (Fig. 2b). GO exhibits compacted and wrinkled layered, clean sheets. This roughness could be attributed to the presence of small Fe_3_O_4_ nanoparticles attached to the GO surface^31–33^.

**Figure 3:**
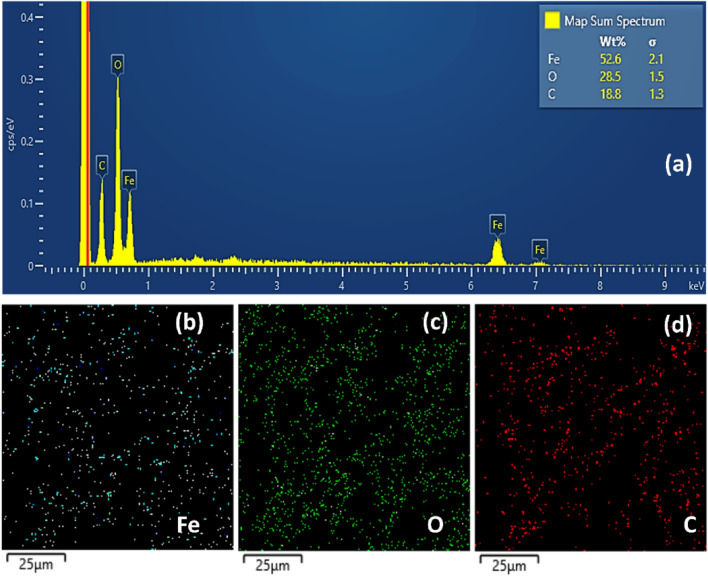
Energy dispersive X-ray pattern of GO@Fe_3_O_4_ (**a**), (**b–d**) elemental mapping of O, Fe and C elements.

The elemental composition was estimated by EDS (Fig. 3a), which indicates Fe (53 wt%), O (28 wt%) and C (19 wt%). The images also show the homogeneous dispersion of Fe on the photocatalyst surface (Fig. 3b) within the analyzed area. Furthermore, carbon and oxygen (Fig. 3d and Fig. 3c) are also dispersed, as expected due to the GO sheets.

**Figure 4:**
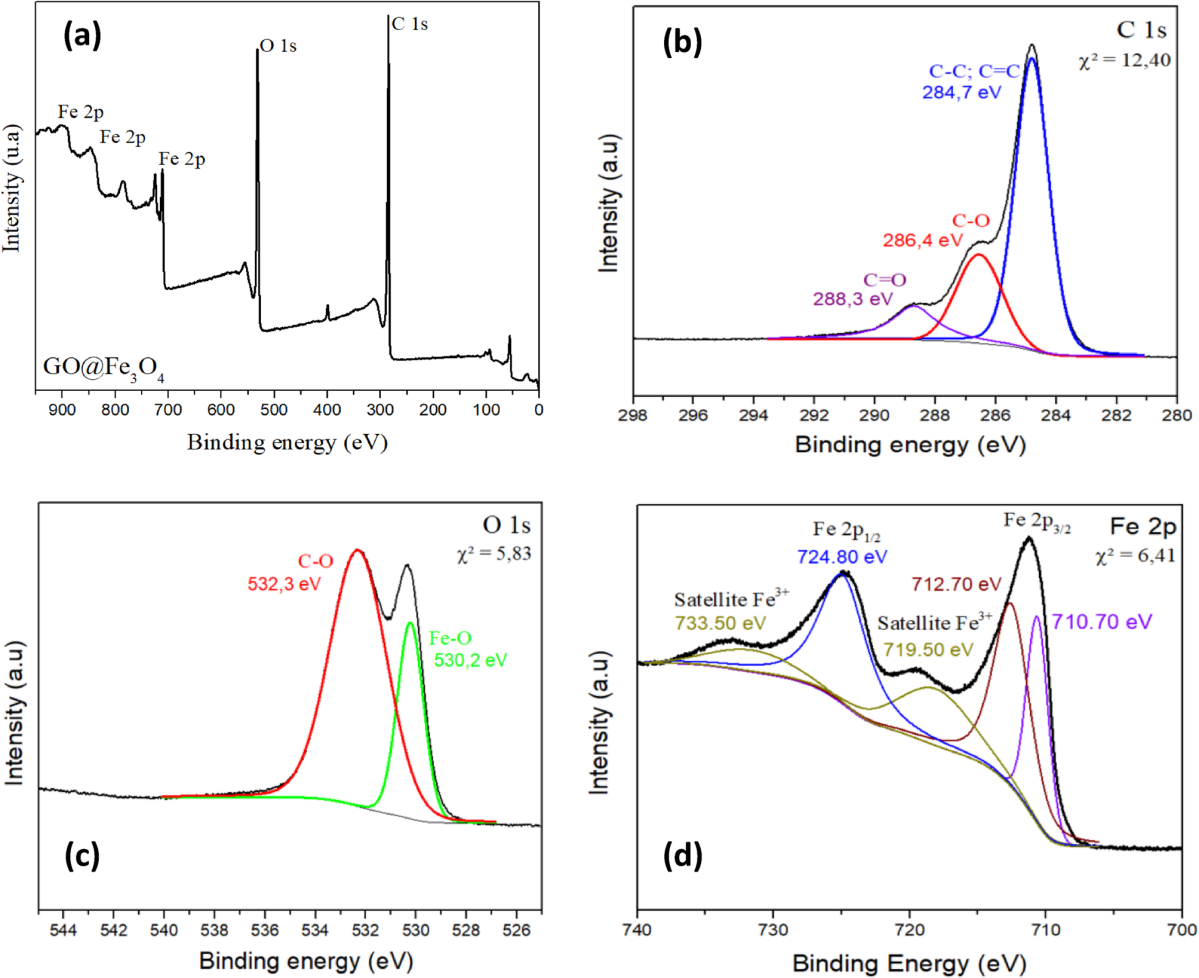
XPS spectra of GO@Fe_3_O_4_ (**a**), C 1 s (**b**), O 1 s (**c**) and Fe 2p (**d**).

The surface chemical properties of GO@Fe_3_O_4_ were investigated via XPS analysis (Fig. 4). The spectra for GO@Fe_3_O_4_ (Fig. 4a) exhibited peaks at the ranges of 250–300 eV and 520–540 eV attributed to C 1 s and O 1 s, respectively. The peaks at the range 710–730 eV, 845 and 900 eV are attributed to the presence of Fe. These results again confirm the purity of the nanomaterial studied in this paper. The high-resolution C 1s spectrum (Fig. 4b) shows peaks at 284.70, 286.40 and 288.30 eV attributed to the presence of non-oxygenated carbon (C–C and C=C), corresponds to sp2 C–C in the aromatic ring, epoxy (C–O) and carbonyl (C=O) groups, respectively. Figure 4b also reveals the highly oxidized state of GO. The O 1 s spectrum (Fig. 4c) can be deconvoluted into two peaks, which were at 530.20 eV, corresponding to Fe–O, and a peak at 532.30 eV attributed to C–O. Another point to note is the absence of a peak corresponding to the Fe–O–C bond in the O 1 s spectrum. The presence of this peak is characteristic of the interaction between magnetite and the oxygenated functional groups present on the surface of graphene oxide. When magnetite is bound to oxygenated groups, this interaction results in a change in the bonding characteristics of the Fe atoms, leading to the formation of the Fe–O–C bond^34^. Moreover, the Fe 2p individual spectrum (Fig. 4d) exhibited peaks at 710.70 eV and 724.80 eV attributed to Fe^2+^ and Fe^3+^ in octahedral sites, respectively, while the peak at 712.70 eV was attributed to Fe^3+^ in tetrahedral sites. These results indicate the presence of Fe_3_O_4_ on the GO surface. Moreover, the satellite peaks at 719.50 and 733.50 eV are attributed to the presence of the Fe^3+^ oxidation state on GO@Fe_3_O_4_^35–39^. These results are in agreement with what is reported in the literature^40^.

The results of the quantitative analysis calculated according to Eq. 2 are presented in Table S2. Analyzing the results from Table S2, it is observed that there is a predominance of iron on the material surface, since the results reported by XPS are representative only of the surface layer of the nanomaterial. Another point to be noted is the difference between the results presented in Table S2, concerning the quantitative analysis of the elements by XPS, and the results obtained by EDS (Fig. 3a). This happens because XPS is an analysis with much lower penetration power than EDS analysis.

**Figure 5:**
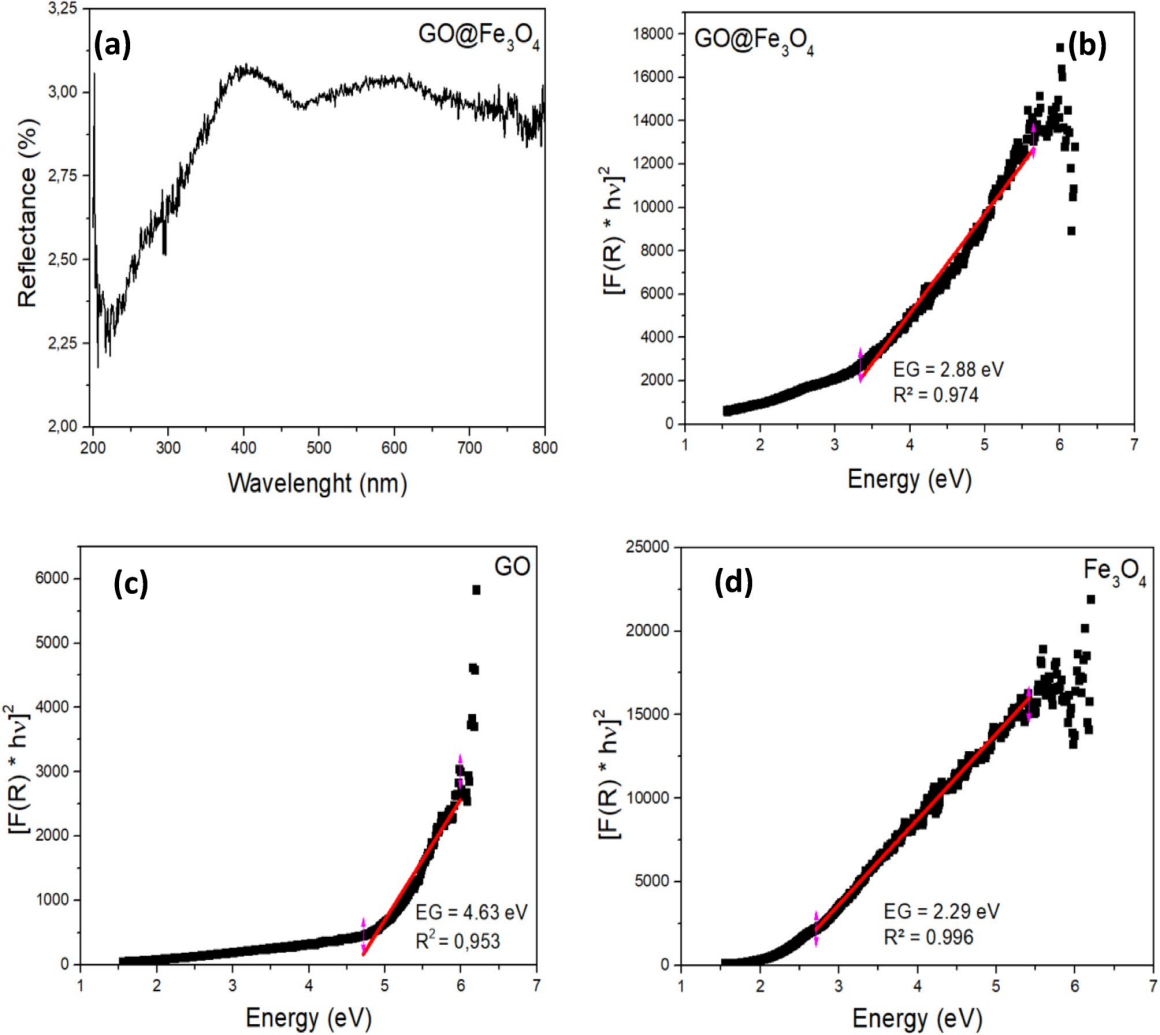
UV‒Vis DRS spectra of GO@Fe_3_O_4_ (**a**), UV‒Vis DRS spectra and Kulbelka–Munk plot [F(R) × hν]^2^ versus hν of GO@Fe_3_O_4_ (**b**), GO (**c**) and Fe_3_O_4_ (**d**).

Figure 5a shows that the material exhibits low reflectance in the UV range, indicating that GO@Fe_3_O_4_ has better photocatalytic activity in this UV region. The band gap energy calculated by the Kubelka–Munk function for GO@Fe_3_O_4_ (Fig. 5b) was found to be 2.88 eV. By comparing these results to the band gap energy for GO (Fig. 5c) and Fe_3_O_4_ (Fig. 5d), it is possible to conclude that the presence of Fe_3_O_4_ leads to narrowing of the band gap in GO. Additionally, it can be speculated that GO@Fe_3_O_4_ may exhibit better photocatalytic activation than its precursors. A low band gap improves the absorption of radiation and, consequently, improves the transfer of electrons from the valence band to the conductive band. Although GO@Fe_3_O_4_ had a greater band gap than its precursor Fe_3_O_4_, the difference was very small. Additionally, GO serves as an acceptor and transporter of electrons, avoiding the recombination of e^−^/h^+^^41–44^.

**Figure 6:**
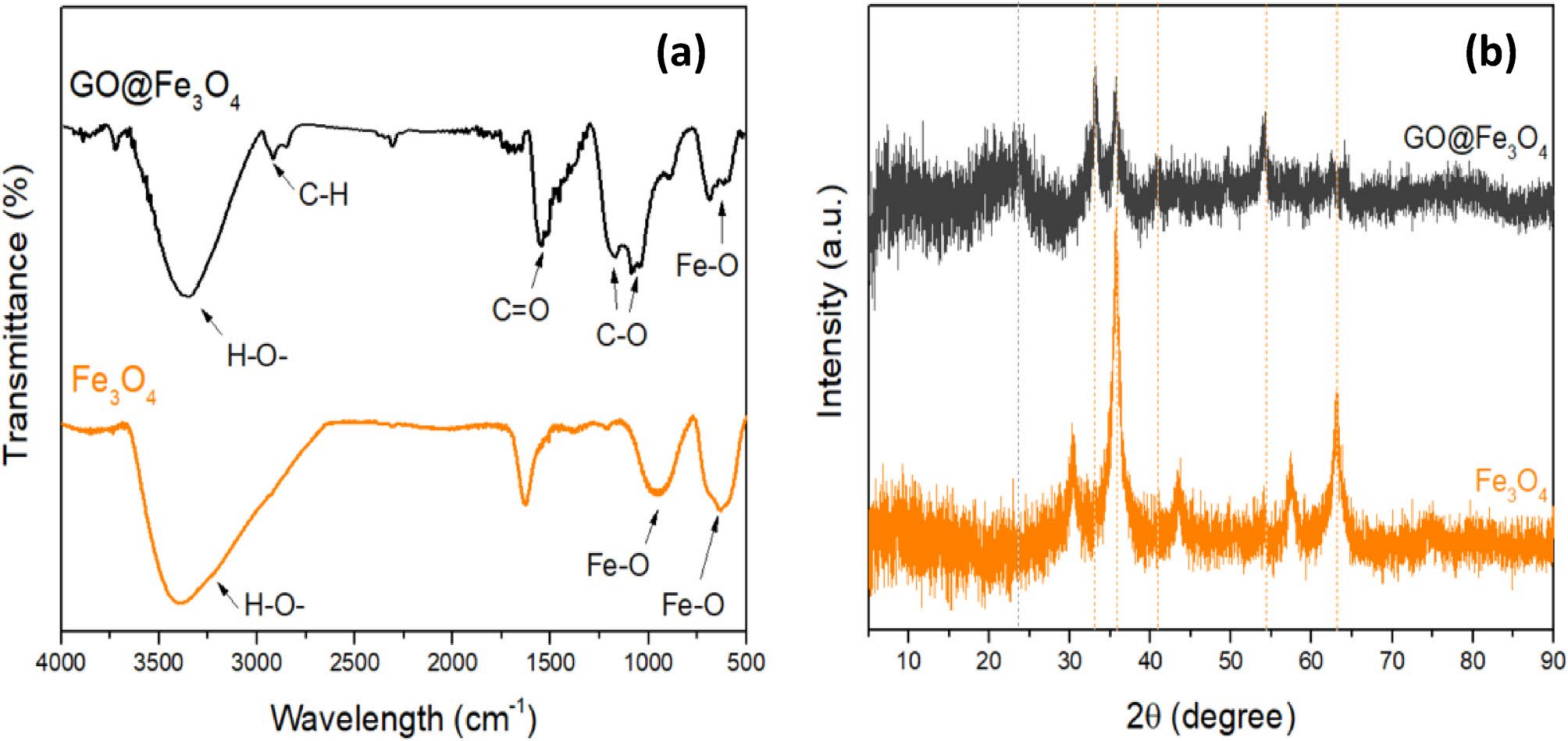
FTIR spectra (**a**) and XRD patterns of Fe_3_O_4_ and GO@Fe_3_O_4_ (**b**).

The FTIR spectra of Fe_3_O_4_ and GO@Fe_3_O_4_ are shown in Fig. 6a where it is possible to observe characteristic peaks of Fe_3_O_4_ in the GO@Fe_3_O_4_ spectra. The peaks at 634.76 and 959.56 cm^−1^ in the Fe_3_O_4_ sample and 590.85 cm^−1^ in the GO@Fe_3_O_4_ spectra is referent to the stretching vibration of the Fe–O bond^31,45^. The band at 1547.80 cm^−1^ in the GO@Fe_3_O_4_ spectrum is attributed to carbonyl groups (–C=O), and the peaks at 1187.43 and 1069.33 cm^−1^ are attributed to the C–O stretching vibrations attributed to the presence of alkoxy and epoxy groups, respectively. The peak at 1629 cm^−1^. The peak at 86 cm^−1^ in the Fe_3_O_4_ spectra is attributed to the bending vibration of the absorbed water. The broad band at 3392.81 cm^−1^ present in the GO@Fe_3_O_4_ and Fe_3_O_4_ spectra are attributed to the stretching of H–O bonds of hydroxyl groups^46,47^.

The XRD patterns of GO@Fe_3_O_4_ and Fe_3_O_4_ are compared in Fig. 6b, in which it is possible to see that the synthesized material exhibits characteristic Fe_3_O_4_ peaks. The broad peak at ~ 2θ 23° (002) for GO@Fe_3_O_4_ is attributed to the partial reduction of GO caused by the co-precipitation reaction of iron ions, strongly alkaline conditions of synthesis, and sample preparation, which requires high temperatures^48,49^. The broad peak at ~ 2θ 23° (002) for GO@Fe_3_O_4_ and the absence of the peak at ~ 2θ 10–11° found in GO diffractograms is a characteristic attributed to the partial reduction of GO caused by the co-precipitation reaction of iron ions, strongly alkaline synthesis conditions, and sample preparation, which requires high temperatures. Similar results have been reported by Chai et al. (2020) and Neolaka et al. (2020), and both studies concluded that the reduction occurred due to the synthesis conditions^50,51^.

The peaks at 2θ 30° (220), 35° (311), 40° (400), 54° (422), 57° (511) and 63° (440) present in the GO@Fe_3_O_4_ and Fe_3_O_4_ spectra are characteristic of Fe_3_O_4_ nanoparticles with a face-centered cubic lattice. The decrease in the intensity of the peaks is attributed to the combination with GO (JCPDS No. 19-0629)^32,41,42,47,48,52,53^.

Figure S1 shows that the material has a zero-point charge of 7.5. The zero-point charge was determined to understand the adsorption and photocatalytic processes since the pH affects the interaction between the material and the target. When the pH < pH_ZPC,_ the GO@Fe_3_O_4_ surface is positively charged; and when the pH > pH_ZPC_ the surface is negatively charged^25^.

**Figure 7:**
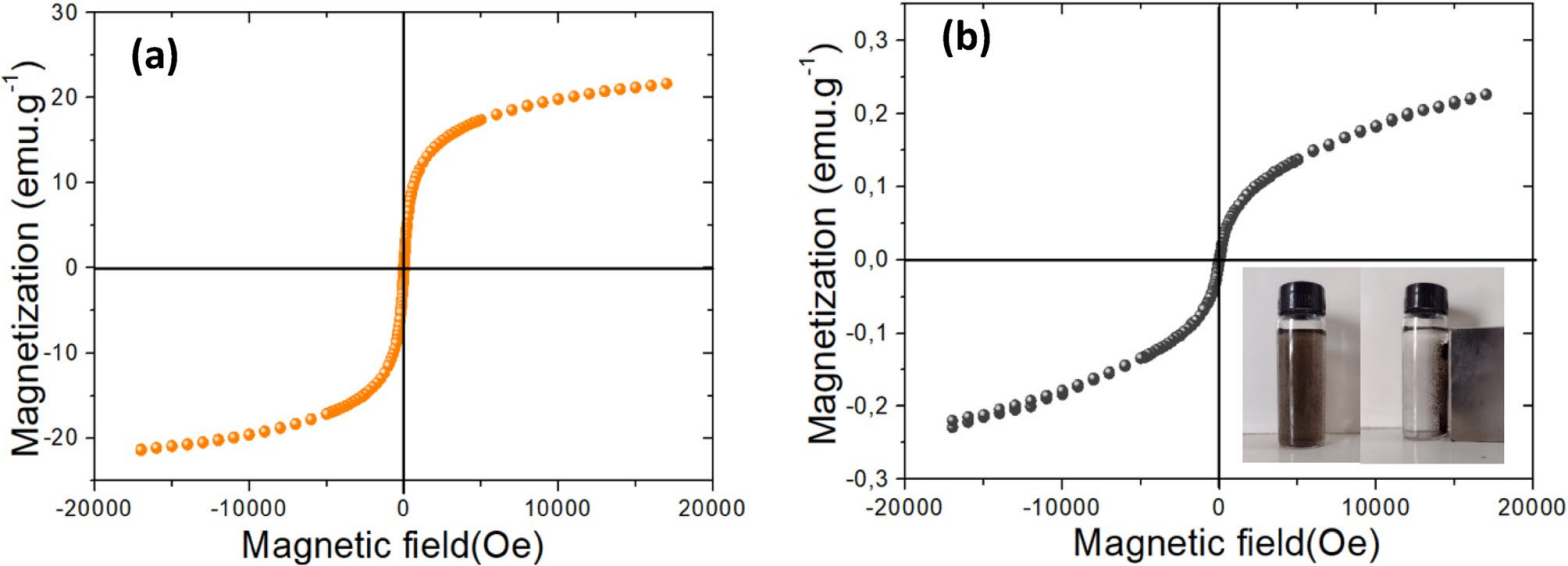
Magnetization hysteresis curves of Fe_3_O_4_ (**a**) and GO@Fe_3_O_4_ (**b**) at room temperature.

Figure 7 shows the magnetization hysteresis curves of Fe_3_O_4_ and GO@Fe_3_O_4_ measured at room temperature, where it can be concluded that the nanocomposite has superparamagnetic properties. This is attributed to the absence of hysteresis and coercivity. In addition, the saturation magnetization for GO@Fe_3_O_4_ and Fe_3_O_4_ are 0.22 emu g^−1^ and 21 emu g^−1^, respectively. The decrease in the saturation magnetization is attributed to the presence of non-magnetic GO, but although the nanocomposite has less saturation magnetization, it was easy to separate the material using a magnetic field, as shown in Fig. 7b^53,54^.

### 2^3^ Factorial design

A 2^3^ factorial design with a central point in triplicate was performed to evaluate the ideal experimental conditions. By analyzing the table of effect estimates (Table S3), it is possible to see that pH was the only parameter significant for the degradation process, with a p-level lower than 5.0% and a 95.0% of confidence interval. Additionally, the effect was negative, indicating that at pH 3, the best results were achieved. This can be explained by the photolytic decomposition of H_2_O_2_ to H_2_O and O_2_, which, is sevenfold greater in basic media than in acid media, and three times greater in neutral media^55,56^.

In addition, these results could be due to the rapid and easy degradation of clonazepam compared with that of other contaminants treated via similar processes^12,57^. Additionally, an increase in the dosage of the nanomaterial leads to an increase in turbidity, which results in a decrease in light transmission and GO@Fe_3_O_4_ activation. Moreover, at high levels, H_2_O_2_ can act as an ^•^OH scavenger, which results in a decrease in the efficiency of the process.

The mathematical model that describes the degradation of clonazepam, which contains significant effects with a 95.0% confidence level in the domain of experimental design, is shown in Eq. (7).

7 $${\text{y}}= 81.67 (\pm 0.43)-4.33 (\pm 0.51){\text{x}}_{1}$$

where y represents the percentage of clonazepam degradation and x_1_ is the pH. The domain of x_1_ is {x ϵ R/ − 1 ≤ x ≤ 1}. The data presented in the ANOVA table (Table S4, supplementary material) showed that there was no evidence of a lack of adjustment for a 95% confidence level because the LFMS/PEMS ratio was lower than the tabulated F_1,8_ (5.32, p-level 0.05). Additionally, the statistical significance of the regression was confirmed because the MSR/MSr ratio was greater than the tabulated F_1,9_ (5.12, p-level 0.05).

These results are confirmed by the surface response (Fig. S2), where it is possible to see that the zone with a higher percentage of degradation is associated with a lower pH. Furthermore, for a fixed pH, the percentage of degradation did not significantly increase when the other associated parameters changed from − 1 to + 1.

Due to the results achieved in the factorial design/surface response analysis, the variable values chosen in the following experiments were 1.0 mg of GO@Fe_3_O_4_, 15.0 mg L^−1^ [H_2_O_2_] with a solution of clonazepam at a pH of 3.

### Adsorption and photodegradation kinetics

**Figure 8:**
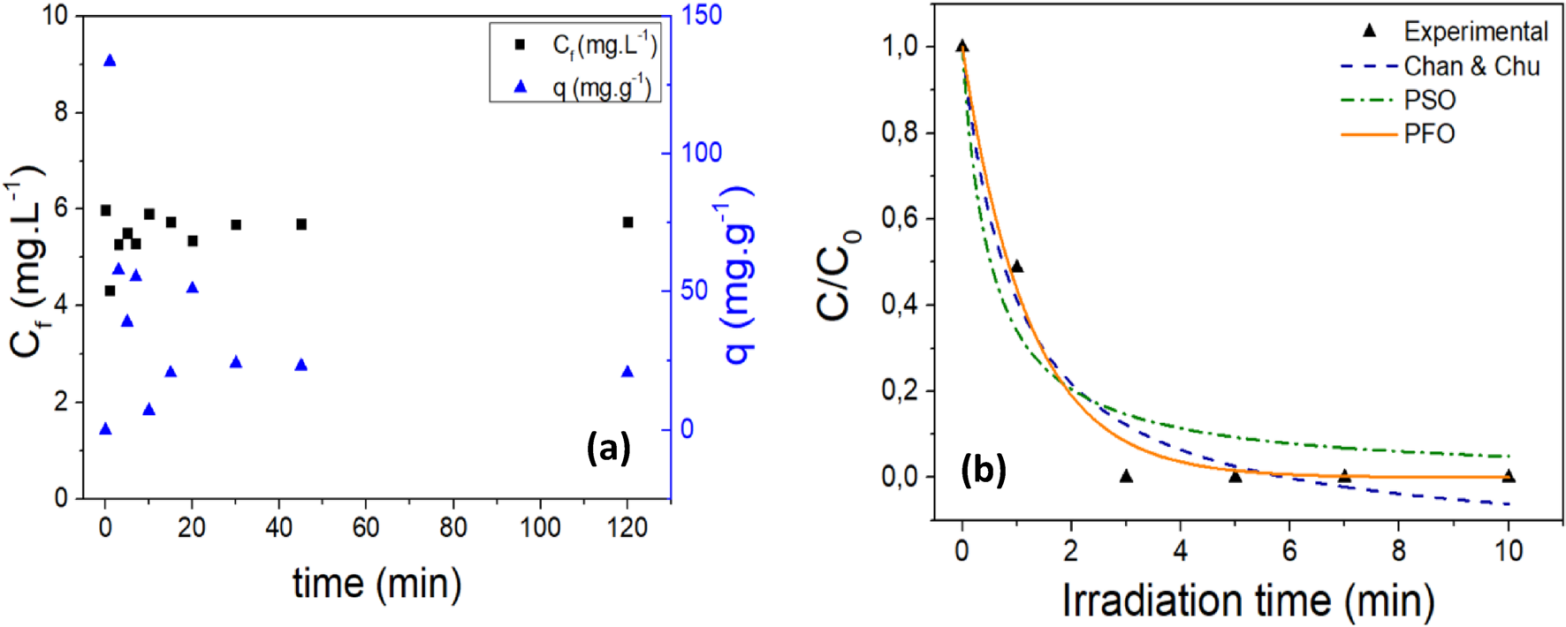
Plot of final concentration of clonazepam and adsorptive capacity evaluation versus irradiation time for the adsorption process (**a**) and photodegradation of clonazepam versus irradiation time ([H_2_O_2_] = 15 mg L^−1^; m_OG@Fe3O4_ = 1.0 mg; pH 3; assisted by UV-C radiation) (**b**).

An adsorption kinetic experiment was conducted to understand and quantify the percentage of CZP removal attributed to the adsorption and photocatalysis processes. Figure 8a and Table S5 (Supplementary Material) depict the results obtained for the adsorption kinetics. The data clearly show that GO@Fe_3_O_4_ was not efficient to fully adsorbing clonazepam molecules. After 1 min of adsorption, the adsorptive capacity reached its peak, indicating faster adsorption of clonazepam; however, this value decreased over the course of the experiment, indicating desorption process. This can be explained considering the pKa of clonazepam, the pH_PZC_ and the medium pH. At pH 3, the surface of GO@Fe_3_O_4_ is positively charged, and as an amphoteric compound that has two dissociation constants, CZP mainly exists in its neutral form since pKa_1_ < pH < pKa_2._ Therefore, nonelectrostatic interactions occur under these conditions^21^.

Another factor that may have influenced the non-adsorption of clonazepam by GO@Fe_3_O_4_ relates to the GO/Fe ratio of the nanomaterial. Since the material contains much more iron than GO, this may have hindered the pollutant adsorption. In the literature, studies can be found where clonazepam was treated by adsorption using material similar to that studied in this work. However, small modifications in synthesis can lead to significant changes in treatment processes^58^. The study conducted by Nascimento et al. (2022) investigated the adsorption of clonazepam by graphene oxide functionalized with iron oxide at a GO/Fe ratio of 1:1. As a result, the authors achieved rapid pollutant adsorption and an adsorption coefficient of 12.326 for the PFO kinetic model^21^.

Figure 8b and Table S6 (Supplementary Material) show the results obtained from the photodegradation kinetic where it is possible to observe that the clonazepam was completely degraded after 5 min. In addition, the HPLC data did not show any other peak, indicating the complete degradation of clonazepam without the formation of any intermediate. Table 1 presents the kinetic parameters, from which it is possible to see that the PFO model better fit the experimental data with a kinetic rate of 0.83 min^−1^.

**Table 1:** Kinetic parameters for the photodegradation process assisted by UV-C radiation.

Model	Parameter		Statistical parameters	
PFO	k_1_ (min^−1^)		R^2^	χ^2^
	0.83 ± 0.09		0.99	0.002
PSO	k_2_ (L mg^−1^ min^−1^)		R^2^	χ^2^
	1.94 ± 0.75		0.93	0.012
Chan & Chu	1/ρ (min^−1^)	σ	R^2^	χ^2^
	1.19	1.17	0.96	0.006

Studies evaluating the degradation of clonazepam are rare; however, it is possible to find works that studied the degradation of pharmaceuticals by similar photocatalysts. Table S7 shows a comparison study of several photocatalysts for the degradation of pharmaceuticals.

Table S7 shows that similar materials were used as photocatalysts for the degradation of different PhACs. The removal percentage was greater than 90.0% for all the reported studies. However, it is well known that the concentration of PhACs in waterbodies is in the range of µg-ng L^−1^^2,58,59^, therefore, it is important to assess the efficiency at which total degradation can be achieved. Table S7 shows that none of these studies achieved 100% degradation, and in some of those studies, the initial and final concentrations were so high that they deviated from real environmental conditions. The efficiency of the process can be improved by the addition of H_2_O_2_ and the use of some radiation source, promoting the formation of radicals. This was not considered in these previous reports.

### Comparison of different processes in clonazepam degradation and radical scavenging tests

**Figure 9:**
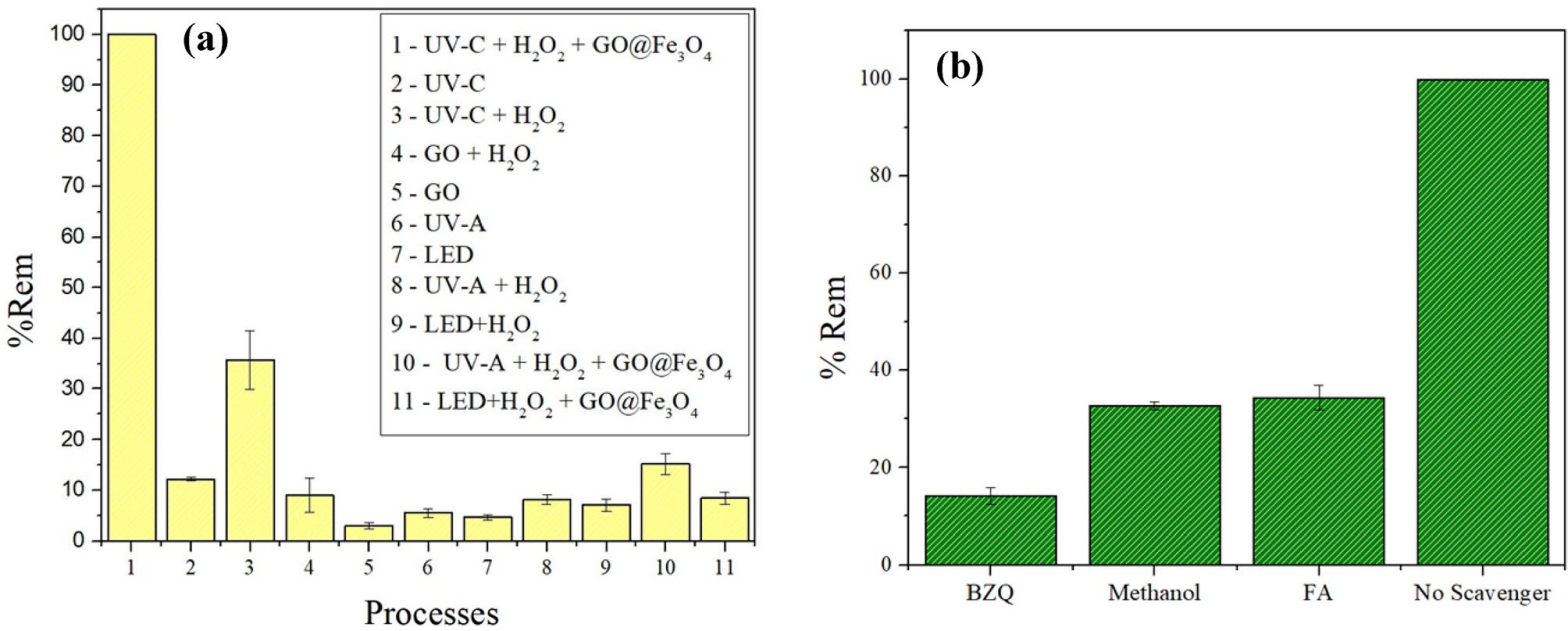
The removal efficiency of clonazepam by different processes (**a**) and scavenging tests (**b**) (pH 3.0; GO and GO@Fe_3_O_4_ dosage = 1.0 mg; [H_2_O_2_] = 15.0 mg L^−1^ and T = 25.0 °C).

The removal efficiency of clonazepam by various processes was studied under identical experimental conditions for 10 min. Figure 9a shows that, compared with other treatment processes, the heterogeneous photo-Fenton process under UV-C radiation results in the highest percentage of clonazepam degradation. Photoperoxidation presented the second-best result (35.73%). These results can be explained by the lower oxidation potential of H_2_O_2_ (1.77 V) compared with the oxidation potential of ^•^OH (2.80 V) and HO_2_• radicals that were generated by the decomposition of H_2_O_2_ under UV-C radiation. Additionally, in the presence of a photocatalyst, other reactions occur, and the generation of radical species is improved, as shown in Eqs. (9) to (11)^49,60^.

A reuse test of GO@Fe_3_O_4_ was conducted, where at the end of the process, the material was recovered and used again in a new process. The results are presented in Figure S3. Analyzing the results in Figure S3, it can be seen that the material was able to degrade the contaminant under the studied conditions even after 5 reuse cycles. SEM and EDS characterization analyses were conducted to examine the material after the process. The results presented in Figures S4 and S5, respectively, showed good stability of the material even after its use. This is seen in the EDS analysis results, where the characteristics found in the material before its use are maintained after the reuse test. Additionally, a small amount of Cl was found. This is justified by the presence of Cl in clonazepam. The Au found in the EDS analysis is due to the sample preparation for the analysis. Furthermore, the SEM images showed the same roughness found in Fig. 2c, referring to the presence of iron oxide on the GO surface.

Moreover, different radiation sources did not result in a high percentage of clonazepam removal, which can be explained by the very efficient source of ^•^OH for each molecule of H_2_O_2_ when irradiated at 254 nm^61^. The UV‒Vis DRS (Fig. 5) spectrum also confirmed the high absorption of radiation in the UV-C range, which resulted in the formation of other radical species. H_2_O_2_ under UV-C radiation produces hydroxyl radicals according to Eq. (8). The degradation observed for the system composed of GO and H_2_O_2_ (5.69%) is attributed to the capacity of GO to act as a catalyst and react with H_2_O_2_ to generate ^•^OH^62^.

8 $${\text{H}}_{2}{{\text{O}}}_{2}+{\text{h}}\upnu\rightarrow 2 {}^{\cdot}{\text{OH}}$$

The degradation of clonazepam under UV-C without the presence of a catalyst and H_2_O_2_ (process number 2), less than 15% shows that although the pharmaceutical was resistant to photolysis, the molecule can absorb UV radiation and decompose^63^.

The radicals scavenging test (Fig. 9b) was performed using benzoquinone (BZQ), methanol and formic acid (FA) to quench superoxide radical ions (${O}_{2}^{\cdot -}$), hydroxyl radical (^•^OH) and holes (h^+^), respectively. The graph shows the efficiency decrease in the presence of the scavengers. In addition, ${O}_{2}^{\cdot -}$ was the main active free radical in the degradation reaction, followed by ^•^OH and h^+^_._ Similar results were obtained by Bashiri and coworkers for the degradation of metronidazole using Fe_3_O_4_/rGO/TiO_2_^64^.

In photo-Fenton reaction, ^•^OH is produced by hydrogen peroxide decomposition under a radiation source and catalyzed by ferrous iron, as shown in Eqs. (9) and (10)^23,65,66^.

9 $${{Fe}}^{2+}+{{H}}_{2}{{O}}_{2}\to {{Fe}}^{3+}+ {{HO}}^{\cdot}+{{OH}}^{-}$$

10 $${{Fe}}^{3+}+{{H}}_{2}{{O}}_{2}\to {{Fe}}^{2+}+{{H}}^{+}+{{HO}}_{2}^{\cdot}$$

The use of some radiation sources also enhances the formation of ^•^OH and the degradation efficiency by the decomposition of complexes that are formed during the conventional reactions, as shown in Eq. (11)^66^.

11 $${{Fe}}{({{OH}})}^{2+}+h\nu \to {{Fe}}^{2+}+{{HO}}^{\cdot}$$

In the homogeneous process, iron ions are consumed, resulting in poor recycling and the formation of iron sludge^18,60,67^. In the heterogeneous photo-Fenton process proposed in this study, the presence of GO helps to accelerate the cycling of Fe(II) and Fe(III) in addition to preventing the aggregation of Fe_3_O_4_ nanoparticles^18,67^.

Thus, the mechanism of degradation occurs with the Fenton reaction and the recycling of Fe(III)/Fe (II) as shown in Eqs. (9) and (10). In addition, the Fe_3_O_4_ nanoparticles anchored in GO could be activated to produce electron and hole pairs. These electrons are transferred to the GO surface, where they hider the recombination of electrons and holes and react with Fe(III) to form Fe(II), which can further react with H_2_O_2_ to form ^•^OH and Fe(III). Moreover, the electron can reduce O_2_ to ${O}_{2}^{\cdot -}$, as shown in Eq. (12). ^•^OH and ${O}_{2}^{\cdot -}$ attack clonazepam molecules until total mineralization. The holes can degrade clonazepam through a direct oxidation mechanism, as described in Eq. (13)^18,67,68^.

12 $${e}^{-}+{{O}}_{2}\to {{O}}_{2}^{\cdot -}$$

13 $${}^{cdot}{{OH}}/{{O}}_{2}^{\cdot -}{/h}^{+}+ clonazepam \to {CO}_{2}+{H}_{2}O+inorganic\, ions$$

**Figure 10:**
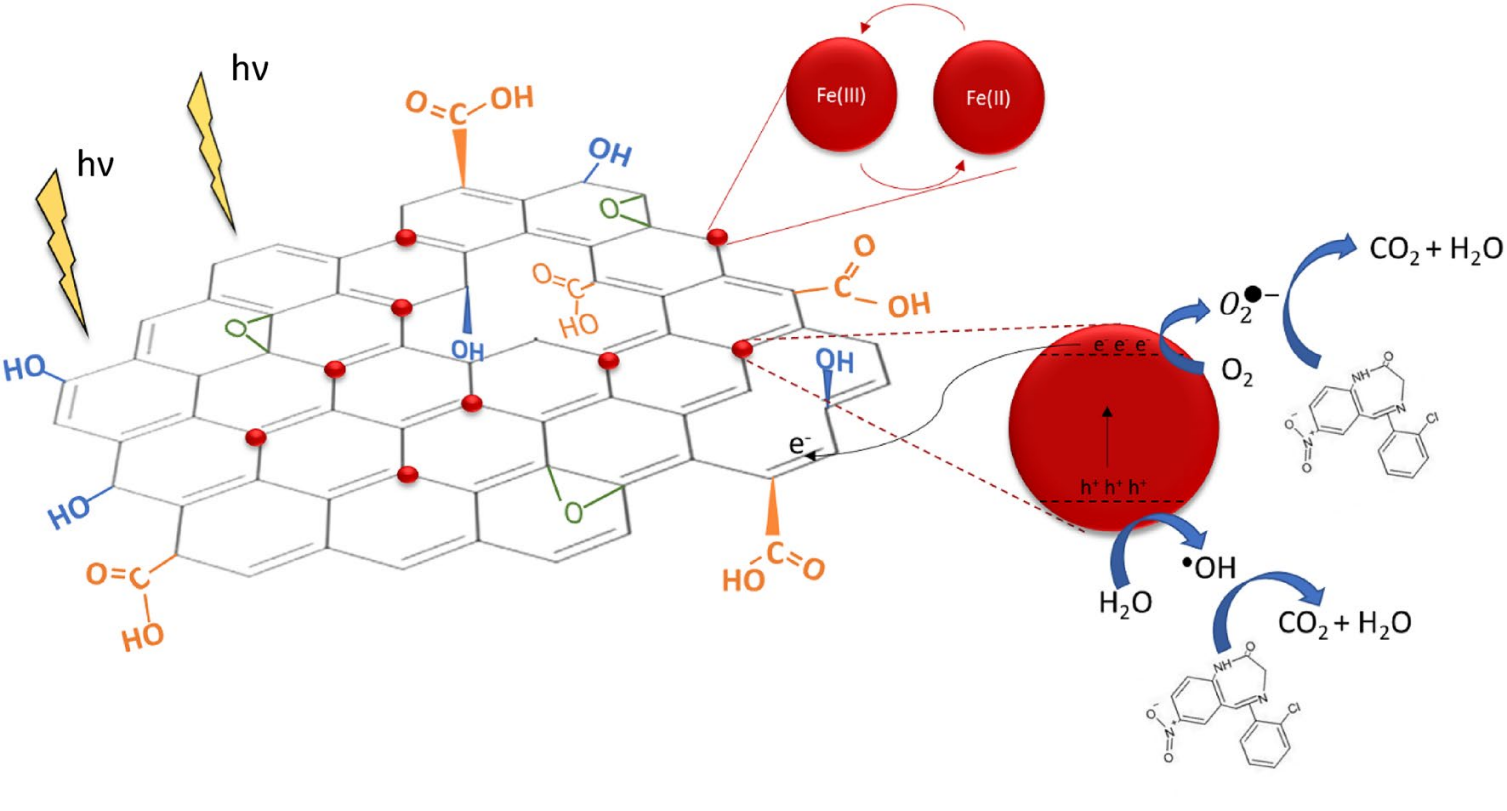
Schematic illustration of clonazepam degradation by GO@Fe_3_O_4_ under UV-C radiation, where  represents the Fe_3_O_4_ on the GO basal plane.

Figure 10 shows a schematic illustration of the proposed mechanism for clonazepam degradation by GO@Fe_3_O_4_ via the photo-Fenton reaction.

## Conclusions

The synthesized graphene oxide-based magnetic photocatalyst exhibited excellent performance for the treatment of water contaminated with clonazepam. Characterization analyses demonstrated the successful functionalization of GO@Fe_3_O_4_. The SEM images comparing the surface morphologies of GO, Fe_3_O_4_ and GO@Fe_3_O_4_ show rough compaction on GO@Fe_3_O_4_, which is evidence of the presence of Fe_3_O_4_ on the GO surface. The SEM image of Fe_3_O_4_ shows octahedral heterogeneous structures attributed to the agglomeration of the particles. The EDS results confirmed the presence of Fe in the composite sample. UV‒Vis DRS spectra show that the material exhibits better radiation absorption in the UV region. Furthermore, the bandgap was 2.88 eV. Although GO enhances the Fe_3_O_4_ bandgap, its presence in photocatalysts is important because GO acts as an electron acceptor and transporter, prevents h^+^/e^−^ recombination and enhances photocatalyst properties. The FTIR spectrum of GO@Fe_3_O_4_ shows a band at 590.85 cm^−1^ attributed to the stretching vibration of Fe–O, confirming the presence of iron oxide in GO. The XRD patterns of GO@Fe_3_O_4_ show peaks from both precursor materials, confirming that functionalization was successful. A 2^3^ factorial design showed that pH was the only factor that significantly affected clonazepam degradation. The adsorption study showed that GO@Fe_3_O_4_ does not adsorb the contaminant. In addition, the photodegradation kinetic showed the complete degradation of a clonazepam solution in 5 min, and the model that better fitted to the experimental data was PFO, for which the kinetic constant was 0.83 min^−1^. The reuse test showed that the material maintains its photocatalytic capacity even after 5 cycles. Furthermore, SEM and EDS analyses conducted after the AOP process showed good stability of the nanomaterial. The heterogeneous photo-Fenton process had the best efficiency when compared to other processes. The proposed mechanism involves the formation of electron and hole pairs by the photoactivation of Fe_3_O_4_ anchored on GO and the transfer of electrons to the GO surface. The generation of ^•^OH occurs by the capture of electrons by H_2_O_2_. Moreover, the electron can react with Fe(III) to form Fe(II), which can further react with H_2_O_2_ to form ^•^OH. The presence of a UV-C irradiation source also improved the formation of ^•^OH. ^•^OH degrades clonazepam to form CO_2_ and H_2_O.

### Supplementary Information

Supplementary Information.

## Data Availability

All the data generated during this study are included in this published article and its Supplementary Information files.

## Abbreviations

AOP: Advanced oxidative processes
CZP: Clonazepam
GO: Graphene oxide
GO@Fe_3_O_4_: Magnetic graphene oxide
HPLC: High-performance liquid chromatography
PFO: Pseudo-first order
PSO: Pseudo-second order
PhACs: Pharmaceuticals active components
