# Supplementary material for: Synthesis of superparamagnetic Fe3O4–graphene oxide-based material for the photodegradation of clonazepam
Source: Sci Rep. 2024 Aug 14;14:18916. doi: 10.1038/s41598-024-67352-8 (PMC11324737; doi:10.1038/s41598-024-67352-8)
Supplement: Supplementary file 1 — Supplementary Information. [file 41598_2024_67352_MOESM1_ESM.docx]

**Supplementary Material**

**Synthesis of super paramagnetic Fe_3_O_4_-graphene oxide-based material for the photodegradation of clonazepam.**

Maryne Patrícia da Silva^1^, Ana Caroline Alves de Souza^1^, Ágata Rodrigues Deodato Ferreira^1^, Pedro Lucas Araújo do Nascimento^1^, Tiago José Marques Fraga^1,2^, Jorge Vinícius Fernandes Lima Cavalcanti^1^, Marcos Gomes Ghislandi^1,3^, Maurício Alves da Motta Sobrinho^1^.

*^1^ Department of Chemical Engineering, Federal University of Pernambuco (UFPE),* *1235 Prof. Moraes Rego Av, Cidade Universitária, zip code: 50670-901, Recife/PE, Brazil*

*^2^ Department of Food Science, Federal University of Pernambuco Agreste (UFAPE), Bom Pastor Avenue, w/n, Boa Vista, zip code: 55292-270, Garanhuns/PE, Brazil*

*^3^ Engineering Campus – UACSA, Federal Rural University of Pernambuco (UFRPE), 300 Cento e sessenta e Três Av., Cabo de Santo Agostinho/PE, Brazil*

**Table S1** 2³ factorial design matrix with central point

| **m_cat_** | **H_2_O_2_** | **pH** |
| --- | --- | --- |
| + | + | + |
| + | + | - |
| + | - | + |
| + | - | - |
| - | + | + |
| - | + | - |
| - | - | + |
| - | - | - |
| 0 | 0 | 0 |
| 0 | 0 | 0 |
| 0 | 0 | 0 |


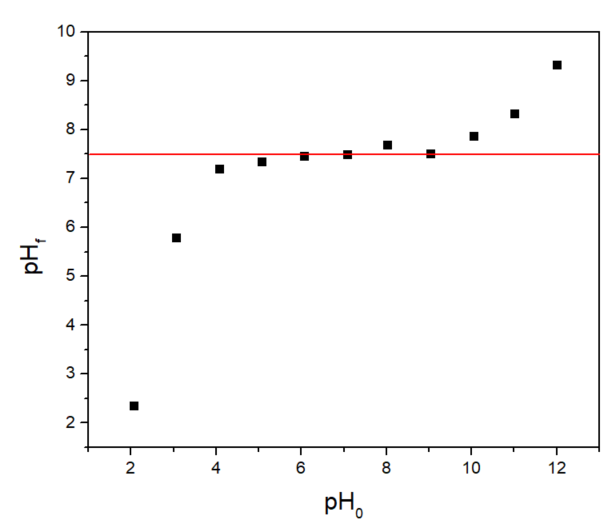


**Fig. S1** pH_ZPC_ of GO@Fe_3_O_4_

**Table S2** Quantitative analysis results by XPS

| Name | Position | RSF | Area | %xi |
| --- | --- | --- | --- | --- |
| C_1S_ | 285.08 | 1 | 1235689 | 5,26 |
| O_1S_ | 532.08 | 2,93 | 1201708 | 15,00 |
| Fe_2p_ | 711.08 | 16,42 | 1001834 | 70,06 |
| Fe_2s_ | 840.08 | 4,57 | 497164,7 | 9,68 |

**Table S3** Estimation of principal and interactions effects of 2³ factorial design.

| **Factor** | **Effect** | **Standard error** | **t (2)** | **p** | **-95%** | **%95%** |
| --- | --- | --- | --- | --- | --- | --- |
| Global average | 81.68 | 0.43 | 189.3557 | 0.000028 | 79.8196 | 83.53133 |
| **Principal effects** |  |  |  |  |  |  |
| [mGO@Fe_3_O_4_](mailto:mGO@Fe3O4) | -0.60 | 1.01 | -0.5956 | 0.611859 | -4.9549 | 3.74992 |
| [H_2_O_2_] | 2.78 | 1.01 | 2.7457 | 0.110991 | -1.5749 | 7.12992 |
| pH | -8.65 | 1.01 | -8.5536 | 0.013394 | -13.0049 | -4.30008 |
| **Two-factor interaction** |  |  |  |  |  |  |
| [mGO@Fe_3_O_4_ x [H_2_O_2_](mailto:mGO@Fe3O4xH2O2)] | 0.67 | 1.01 | 0.6599 | 0.577166 | -3.6849 | 5.01992 |
| [mGO@Fe_3_O_4_ x pH](mailto:mGO@Fe3O4xpH) | -1.99 | 1.01 | -1.9697 | 0.187687 | -6.3449 | 2.35992 |
| [H_2_O_2_] x pH | 1.39 | 1.01 | 1.3716 | 0.303782 | -2.9649 | 5.73992 |
| **Three-factor interaction** |  |  |  |  |  |  |
| [mGO@Fe_3_O_4_ x [H_2_O_2_] x pH](mailto:mGO@Fe3O4xH2O2xpH) | 3.93 | 1.01 | 3.8826 | 0.060390 | -0.4249 | 8.27992 |

**Table S4** Analysis of variance (ANOVA) of process variables for factorial design 2^3^ with central point in triplicate, pure error and 95% confidence.

| **Source of variation** | **SS** | **Df** | **MS** | **F** | **p** |
| --- | --- | --- | --- | --- | --- |
| Regression | 149.73 | 1 | 149.73 | 21.03 | 0.0013 |
| residual | 64.06 | 9 | 7.12 |  |  |
| Lack of fit | 0.28 | 1 | 0.28 | 0.036 | 0.86 |
| Pure error | 63.78 | 8 | 7.97 | 18.78 | 0.0025 |
| Total | 213.8 |  |  |  |  |
| Multiple R | 83.69 |  |  |  |  |
| Multiple R² | 70.03 |  |  |  |  |

Where Df = Degree of freedom; SS = Sum of squares; MS = Mean square

**(b)**

**(a)**


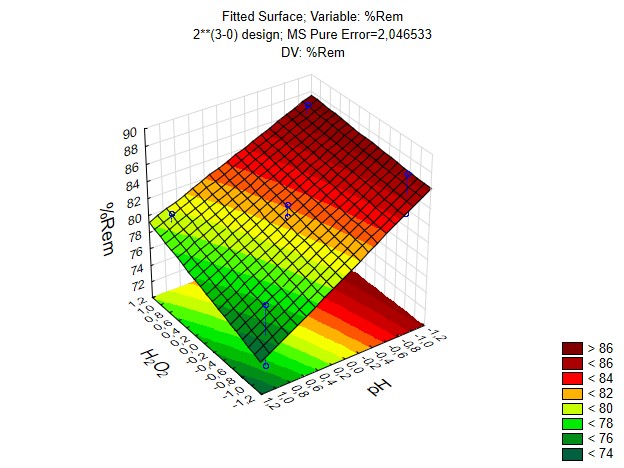

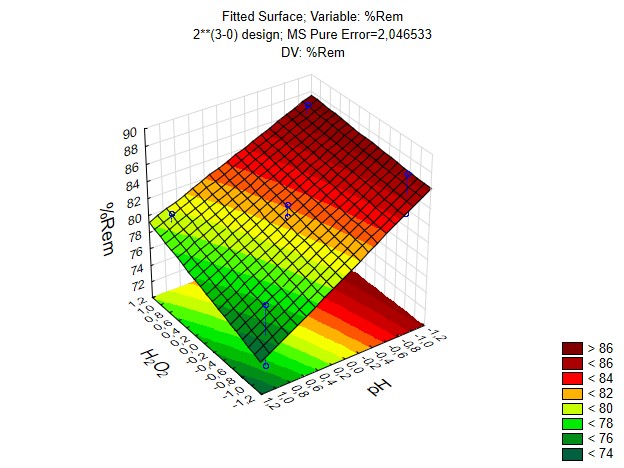

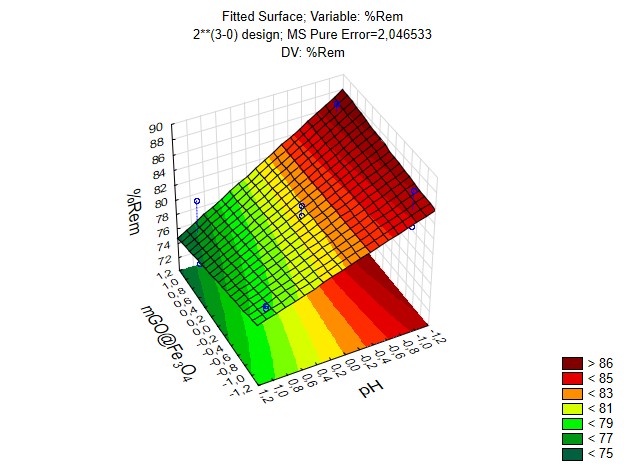

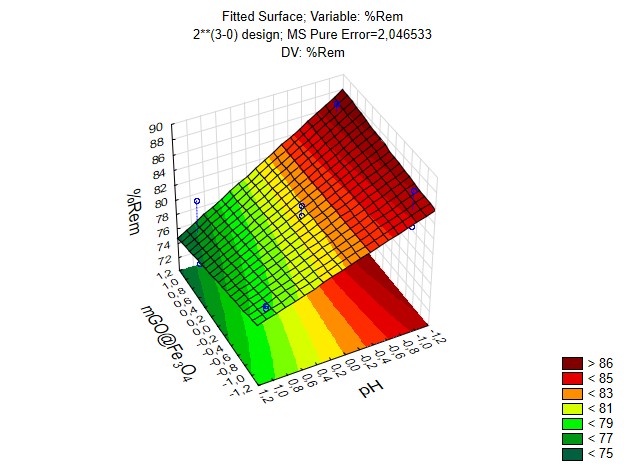


**Fig. S2** Surface response graphs for percentage of clonazepam degradation (% Rem) for [H_2_O_2_] versus pH (**a**) and mGO@Fe_3_O_4_ versus­ pH (**b**)

**Table S5** Final concentration and adsorptive capacity evaluation *versus* time for adsorption process

| **t (min)** | **C_1_** | **C_2_** | **C_MED_** | **%Rem** | **q (mg/g)** |
| --- | --- | --- | --- | --- | --- |
| 0 | 6.12 | 5.86 | 5.99 | 0.00 | 0.00 |
| 1 | 4.53 | 4.11 | 4.32 | 27.89 | 133.64 |
| 3 | 5.53 | 5.00 | 5.27 | 12.09 | 57.93 |
| 5 | 5.60 | 5.41 | 5.50 | 8.15 | 39.07 |
| 7 | 5.49 | 5.11 | 5.30 | 11.56 | 55.41 |
| 10 | 6.03 | 5.78 | 5.90 | 1.48 | 7.11 |
| 15 | 5.73 | 5.73 | 5.73 | 4.32 | 20.69 |
| 20 | 5.52 | 5.19 | 5.35 | 10.66 | 51.07 |
| 30 | 5.71 | 5.66 | 5.69 | 5.06 | 24.27 |
| 45 | 5.83 | 5.57 | 5.70 | 4.87 | 23.36 |
| 120 | 5.42 | 6.04 | 5.73 | 4.33 | 20.74 |

**Table S6** Results of photodegradation kinetics

| **t (min)** | **C_1_** | **C_2_** | **C_MED_** | **C/C_0_** |
| --- | --- | --- | --- | --- |
| 0 | 6.13 | 5.98 | 6.05 | 1 |
| 1 | 2.97 | 2.95 | 2.96 | 0.49 |
| 3 | 0.00 | 0.00 | 0.00 | 0 |
| 5 | 0.00 | 0.00 | 0.00 | 0 |
| 7 | 0.00 | 0.00 | 0.00 | 0 |
| 10 | 0.00 | 0.00 | 0.00 | 0 |

**Table S7** Studies of pharmaceuticals degradation using GO functionalized with Fe_3_O_4_

| **PhAC** | **System** | **Dosage of the photocatalyst/PhAC volume** | **Initial concentration** | | **pH** | **Time** | **Model** | **%Rem** | **Ref.** |  |
| --- | --- | --- | --- | --- | --- | --- | --- | --- | --- | --- |
|  |  |  |  |  |  |  |  |  |  |  |
| Chlorpheniramine | GO-Fe_3_O_4_/H_2_O_2_ | 2 g.L^-1^ | 120 mg/L | 5.0 | | 60 min | PSO | 96.00 | ^50^ |  |
| Carbamazepine | rGO_(10wt%)_-Fe_3_O_4_/sun light | 0.5 g.L^-1^ | 5 mg/L | 6.5 | | 180 min | PFO | 98.7 | ^51^ |  |
| Tetracycline | α-Fe_2_O_3_/rGO/ LED | 5 g.L^-1^ | 5 mg/L | N/I | | 140 min | PFO | 99.0 | ^52^ |  |
| Ibuprofen | α-Fe_2_O_3_/rGO/LED | 5 g.L^-1^ | 5 mg/L | N/I | | 140 min | PFO | 93.0 | ^52^ |  |
| Tetracycline hydrochloride | Fe_3_O_4_-rGO_5wt%_/H_2_O_2_ | 0.5 g.L^-1^ | 60 mg/L | 4.6 | | 90 min | PFO | 98.11 | ^53^ |  |
| Clonazepam | [GO@Fe_3_O_4_/H_2_O_2_/UV-C](mailto:GO@Fe3) | 0.12 g.L^-1^ | 6 mg/L | 3.0 | | 5 min | PFO | >95% | This Work |  |

*N/I: not informed


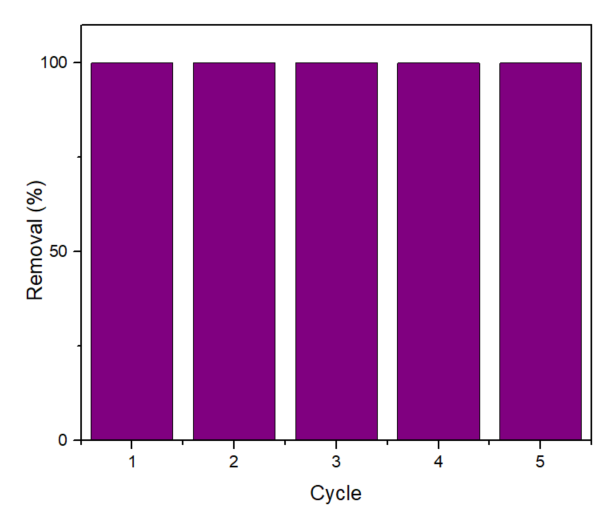


**Fig S3** GO@Fe_3_O_4_ reuse cycles


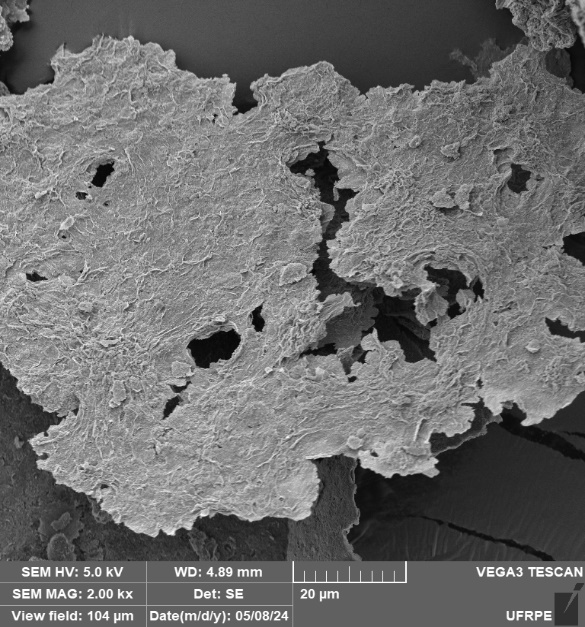

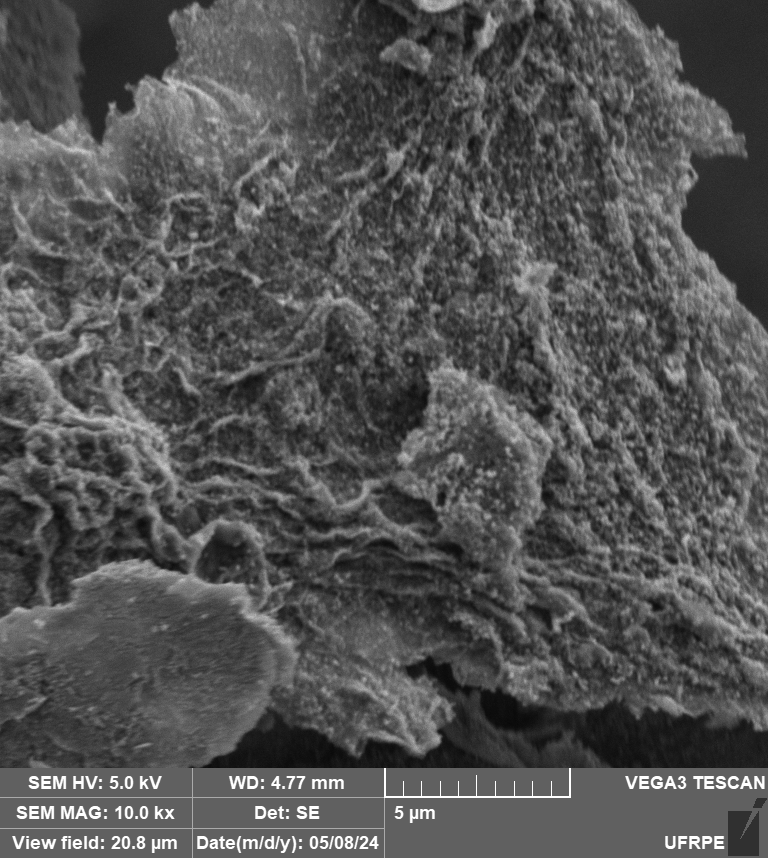


5µm

20µm

**Fig. S4** SEM images of GO@Fe_3_O_4_ after AOP process

**(a)**

**(d)**

**(c)**

**(b)**


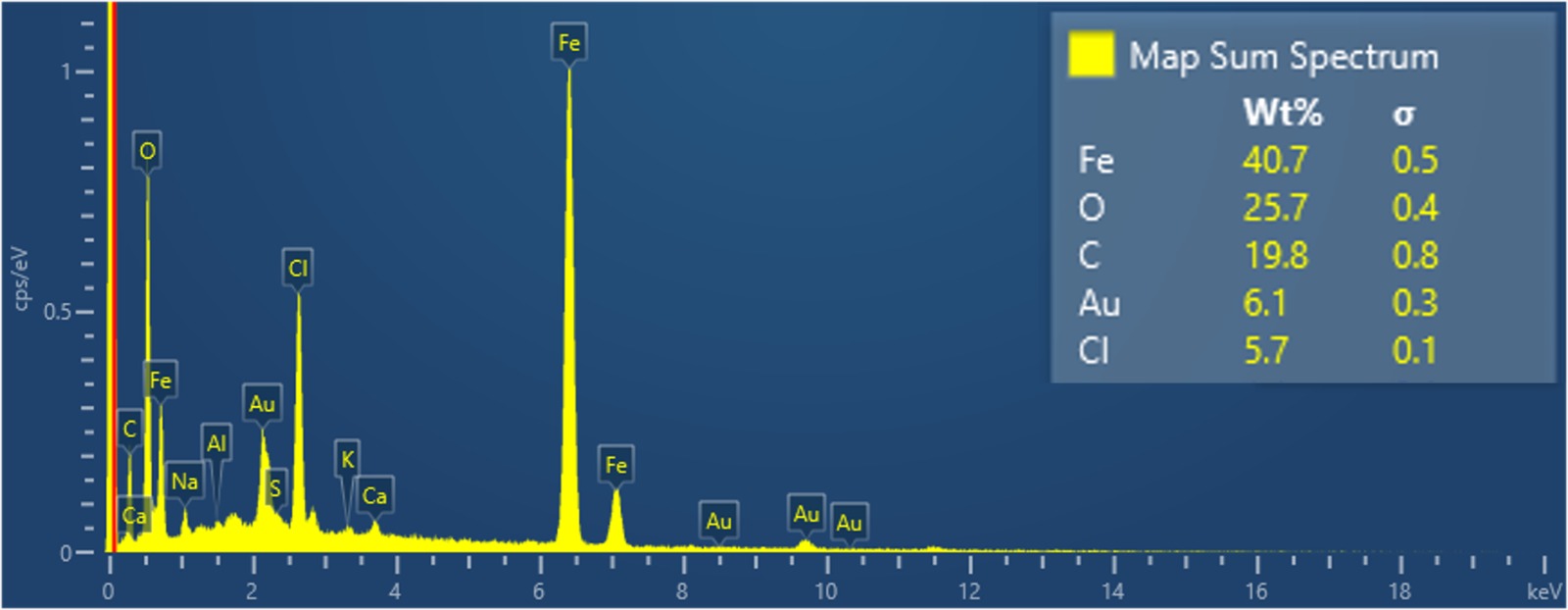

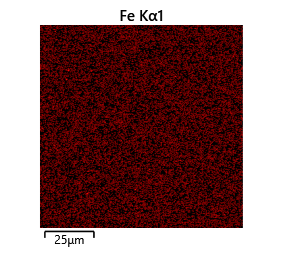

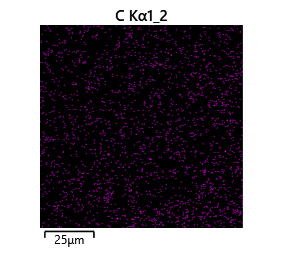

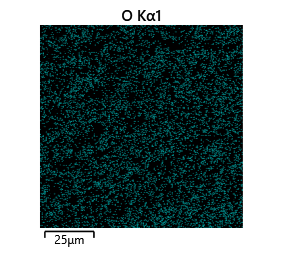


**Fe**

**C**

**O**

**Fig. S5** Energy dispersive X-ray pattern after AOP process of GO@Fe_3_O_4_ **(a), (b-d)** elemental mapping of Fe, C and O elements.
